# Barriers to community-based drug dependence treatment: implications for police roles, collaborations and performance indicators

**DOI:** 10.7448/IAS.19.4.20879

**Published:** 2016-07-18

**Authors:** Yi Ma, Chunhua Du, Thomas Cai, Qingfeng Han, Huanhuan Yuan, Tingyan Luo, Guoliang Ren, Gitau Mburu, Bangyuan Wang, Olga Golichenko, Chaoxiong Zhang

**Affiliations:** 1 Yuxi CDC, Yuxi City, Yunnan, China; 2 AIDS Care China, Kunming, Yunnan, China; 3 International HIV/AIDS Alliance, Brighton, UK; 4 Department of Health Research, Lancaster University, Lancaster, UK; 5 Department of Anthropology, Washington University in St. Louis, MO, USA

**Keywords:** community-based treatment, drug use, policy, harm reduction, police, China

## Abstract

**Introduction:**

Worldwide, people who use drugs (PWUD) are among the populations at highest risk for HIV infection. In China, PWUD are primarily sentenced to compulsory detainment centres, in which access to healthcare, including HIV treatment and prevention services, is limited or non-existent. In 2008, China's 2008 Anti-Drug Law encouraged the development and use of community-based drug dependence rehabilitation, yet there is limited evidence evaluating the efficacy and challenges of this model in China. In this study, we explore these challenges and describe how cooperation between law enforcement and health departments can meet the needs of PWUD.

**Methods:**

In 2015, we conducted semi-structured, in-depth interviews with all four staff members and 16 clients of the Ping An Centre No. 1 for community-based drug treatment, three local police officers and three officials from the local Centre for Disease Control. Interviews explored obstacles in implementing community-based drug dependence treatment and efforts to resolve these difficulties. Transcripts were coded and analyzed with qualitative data analysis software (MAXQDA 11).

**Results:**

We identified three challenges to community-based drug treatment at the Ping An Centre No. 1: (1) suboptimal coordination among parties involved, (2) a divergence in attitudes towards PWUD and harm reduction between law enforcement and health officials and (3) conflicting performance targets for police and health officials that undermine the shared goal of treatment. We also identified the take-home methadone maintenance treatment model at the Ping An Centre No. 1 as an example of an early successful collaboration between the police, the health department and PWUD.

**Conclusions:**

To overcome barriers to effective community-based drug treatment, we recommend aligning the goals of law enforcement and public health agencies towards health-based performance indicators. Furthermore, tensions between PWUD and police need to be addressed and trust between them fostered, using community-based treatment centres as mediators. The preliminary success of the take-home methadone maintenance treatment pilot can serve as an example of how collaboration with the police and other government agencies can meet the needs of PWUD and contribute to the success of community-based treatment.

## Introduction

In the 1980s and 1990s, China experienced a surge in drug use, predominantly involving heroin, in which the number of registered drug users increased more than 15-fold between 1990 and 2004 [1]. Coincidentally, China's HIV epidemic began in earnest when an outbreak among people who use drugs (PWUD) was documented in Yunnan Province in 1989, a key region for drug trafficking as well as subsequent HIV spread [2]. By 2012, the prevalence of HIV among PWUD was estimated at 10.1% [3], and the number of new cases of HIV transmission due to injection drug use was estimated at 28.4% [4]. Compared to the national rate of HIV infection (<0.1% in adults) [4], HIV infection rates among PWUD are extremely high and PWUD continue to be among those at highest risk for HIV infection in China [5,6].

In response to the re-emergence of drug use in the 1980s, the Chinese government launched a policy under which PWUD are sentenced to compulsory detention and labour centres, where detainees are subjected to punitive measures [7]. Studies have shown that in these centres, PWUD generally receive little or no access to HIV prevention, education or medical treatment (including antiretroviral therapy for HIV-positive detainees) [7]. There is significant evidence showing that compulsory detention is ineffective at reducing drug use, production and trafficking in Asia [8]. In addition, “treatment” in some, but not all, compulsory detention centres in China has been associated with increased prevalence of HIV risk factors among detained PWUD [9,10].

Subsequently, in June 2008, the Chinese government introduced a new drug control law that emphasized a “human-centred” approach and stressed treatment through community-based drug dependence treatment (CDDT). One of the key interventions of the CDDT programme is to provide methadone maintenance treatment (MMT), which uses daily administered methadone over a prolonged period of time (possibly indefinite) as treatment for someone who is dependent on opioids. MMT was established as a response to the HIV/AIDS epidemic, fuelled by needle-sharing among PWUD [3,11]. The goal of MMT is to reduce drug-related harm as well as HIV risk through needle-exchange programmes, outreach and access to HIV testing [12]. However, there are fundamental challenges that still remain, some involving interactions between local police and PWUD. By law, CDDT facilities are to be implemented by sub-district administrative agencies with the help of local police (the Public Security Bureau, PSB) and health departments, but in practice the PSB plays a dominant role in enforcing drug control laws. Police have the sole authority to refer PWUD to compulsory detoxification or CDDT. Successful MMT is a challenge due to the required daily clinic visits, and this challenge is further complicated by frequent arrests made in and around MMT clinics by local police [13], which prevent access and adherence to MMT and interrupt HIV prevention among MMT participants. Furthermore, PWUD must register with the police to access take-home methadone, after which the police monitor PWUD through regular, obligatory meetings that may entail urine testing for drug use.

Another challenge for China's CDDT remains to be addressed: the infrastructure of “community” is largely absent in Chinese people's social and political lives [14], and thus cooperation with higher-level government agencies, including the police force, to support community-based treatment has lagged [13]. As a result, CDDT centres have limited focus on patient-centred approaches such as psychoeducation, treatment literacy, motivational counselling, community outreach, and peer-based case management, all of which are known to reduce relapse rates [15–17]. To address the concerns of the government and other stakeholders regarding the development, organizational structure, staffing and efficacy of CDDT [18], in 2015 the government launched the CDDT work plan for 2016 to 2020, to further develop the CDDT model through pilot programmes. With this new work plan, there is an emerging need to analyze existing pilot programmes to understand the challenges to effective CDDT and to identify strengths that can be implemented on a larger scale or at multiple locations.

To this end, investigated the Ping An Centre No. 1 project, a pilot CDDT programme established in late 2013 and implemented through collaborative efforts between the Hongta District PSB, the Hongta District Centre for Disease Control (CDC) and AIDS Care China (ACC), a local Chinese non-governmental organization. The Ping An Centre No. 1 provides support for drug dependence treatment (health examinations, treatment referrals, urine testing, naloxone for overdose prevention, MMT), social and educational activities, vocational training, psychological counselling and related HIV and Hepatitis C virus prevention and treatment, in addition to providing facilities for cooking, laundry and showering. As a result of collaboration between ACC, the CDC and PSB, take-home MMT was piloted. ACC initiated and organized meetings to facilitate discussion and agreement between the PSB and CDC on the standard procedures of the pilot, and each entity plays a role in this service. ACC designed the programme, while CDC and PSB implement and manage it. The PSB deals with the legal issues around taking home methadone and is responsible for checking the criminal records of the clients who apply to take methadone home and approving these who qualify according to the standards agreed upon with the CDC.

In this study, we used semi-structured interviews to understand the police's views on PWUD and drug use, as well as how PWUD and CDC staff feel about the police. We identify challenges of CDDT associated with the role of the police and examine how the role of police in collaboration, approach and performance indicators could contribute to these challenges and how changes in these might improve CDDT.

## Methods

### Study setting

This study was conducted in Yuxi City, in Yunnan Province. Yunnan Province has one of the highest rates of drug use and HIV infection in China [1]. In 2013, 5027 registered PWUD lived in Yuxi City and 1905 lived in the broader Hongta District. Over 80% of the city's registered PWUD have been arrested for heroin use. The Ping An Centre No. 1 was established in late 2013 by the Hongta District CDC in collaboration with ACC. This programme was officially approved by the Yuxi City-level health bureau and CDC and was supported by the Hongta District PSB, Department of Justice, and District Drug Control Office.

### Study design

After eight months of ethnographic study (February to October 2015) in Ping An Centre No. 1 to develop a relationship of trust with potential informants, we conducted semi-structured interviews to examine the attitudes, perspectives and interactions of the staff of Ping An Centre No. 1, the PSB (the local police), the CDC, and PWUD served by the centre (October 2015 and December 2015).

### Participant recruitment

Participants were selected purposely to represent perspectives from the different stakeholders [19] who were involved in local drug control and harm reduction efforts. We interviewed four study groups: treatment centre clients (*n=*16; 12 males and 4 females), all treatment centre staff (*n=*4), local police officers (*n=*3), and officials from the local CDC (*n=*3). For the PWUD, participation was based on age (>18 years) and the duration of enrolment. To capture the perspectives of PWUD who had varied experiences at the centre, all participants (*n=*254, of whom 229 were male and 25 were female) were stratified into four groups according to the duration of time in which they had accessed services at the centre: 0 to 3 months, 4 to 6 months, 6 to 12 months and 13+ months. Participants were then recruited based on a gender-proportional method, and selected based on willingness to participate. For the PSB, only three of a total of seven police officers agreed to be interviewed; for the CDC, the interviewed respondents represented all staff in the local CDC centre. All participants were required to consent to participation, after being informed that they had the right to withdraw at any stage of the interview.

### Data collection

Interviews were conducted in Chinese in private settings to protect participants’ privacy. Each interview took 45 to 60 minutes. We collected information on participants’ views and understanding of drug use, anti-drug policies and the community-based structure of the Ping An Centre No. 1, as well as participant views on the different groups involved in this study, the goals and agendas of the different groups involved and cooperation among these groups.

### Data analysis

Interview data were transcribed and translated to English. Transcribed interviews were then coded using a scheme developed according to the signification of concepts, beliefs, topics, and terms that emerged from the interviews. Codes and concepts were then constantly compared [20] using MAXQDA 11 for occurrence and co-occurrence patterns and emergent themes [20,21].

### Ethical considerations

This study was conducted in keeping with ethical guidelines related to human participants [22]. Participation was entirely voluntary and informed oral consent was obtained from all participants. Apart from light refreshments during the interviews, no other incentives were provided. The Institutional Review Board of Washington University in St. Louis approved this study in December 2014.

## Results

Three major themes emerged in our interviews with Ping An Centre No. 1 staff, centre clients, the PSB and CDC: (1) suboptimal coordination among the parties involved in CDDT, (2) a divergence in attitudes held by the PSB and CDC towards harm reduction and PWUD and (3) conflicting performance targets of the police and the health officials that undermined the shared goal of treatment. In addition to these themes, we identified preliminary evidence from the Ping An Centre No. 1 project regarding the elements key to successful implementation of CDDT.

### Suboptimal coordination and collaboration between the police and other stakeholders

Officials from the CDC, local police and PWUD identified an urgent need for improved coordination and collaboration both among governmental agencies and between governmental agencies and the community. Although a certain level of collaboration exists, a CDC interviewee expressed a specific need for collaboration with police:

As a health department, we can take responsibility for the health problems of PWUD, such as HIV and hepatitis C, as well as methadone treatment. But if they break the law … for example, last year one person brought a knife to the clinic and asked for methadone, can we deal with that? No! That's why we need cooperation with the police.

Although the national policy explicitly recommends multi-agency cooperation to successfully implement community-based treatment, local police officers implied that existing collaboration is suboptimal. They expressed the need for increased coordination and unified direction to balance the aspiration of the police to maintain peace, reduce crime and reduce drug use on the one hand, with the need to rehabilitate PWUD on the other:

I'm only a police officer. We do not have the right to ask other agencies for cooperation or tell them what they need to do. If there were an independent NNCC [National Narcotics Control Commission] work team, the situation would be much better, and multi-agency cooperation could become workable.

This point was particularly important because interviewed police officers implicitly welcomed a shift from detention and punitive approaches to voluntary community-based approaches, yet they felt that this was contradictory to existing goals within the force. Police questioned the effectiveness of the compulsory approach, but it was apparent that the vision and goals of voluntary community-based treatment were not widely understood within the police force:

We are so tired of repetitively arresting and releasing the same drug users. Compulsory rehabilitation is useless and very expensive. The government has to pay a lot of money for it. This job is very dangerous; many colleagues have gotten injured [by PWUD]. We are also seeking more effective approaches. But no one knows how to do that.

This sentiment of lack of direction, coordination and common understanding among the police and other government agencies in general was echoed by a CDC official, who stated, “if the leaders had the correct understanding [and gave the right orders], other things would be easy.”

Although we identified recurring themes of fear of police by PWUD (see below), PWUD also recognized the role of police and the necessity for cooperation, as well as their own role in collaboration for effective community-based treatment:

It is impossible for me to trust them [police] suddenly. Trust needs a long time [to build]. But they are necessary; we need their approval [for piloting this treatment model]. Cooperation is [therefore] the most ideal [solution]. I really hope I can accomplish it [CDDT] here [at the Ping An Centre No. 1].

In all, many informants identified a distrust of local police but recognized the necessity of collaboration. All informants expressed that they felt they could trust the Ping An No. 1 treatment centre in their interviews.

### Divergence in attitudes towards PWUD and harm reduction held by the PSB and CDC

One major challenge was a divergence in understanding of drug use and PWUD among different stakeholders. Many police viewed PWUD as people who constantly threatened social order. Given the general police mandate of maintaining the peace, it was not surprising that they felt that PWUD should be isolated through compulsory detention. Justifying the need for such an approach, a police official stated the following:

In general, the police make judgements based on people's behaviour. They [PWUD] are often involved in unlawful activities or even commit crimes, you know, like stealing, robbing and selling drugs.

Although PSB often adopted this view, criminalizing PWUD, most CDC officials considered PWUD as “patients with drug dependence problems” or belonging to a “high-risk population who needed public health intervention” and therefore they adopted harm reduction approaches:

We see this group of people as a high-risk population with a disease. To crack down on PWUD sometimes even forces them to take riskier behaviours. The concept of harm reduction stresses [their] humanity. [We need] to view them not as criminals, but human beings with weaknesses and needs.

### Conflicting performance targets of the PSB and CDC

The divergent understandings were reinforced by the conflicting agendas of the PSB and CDC. Performance targets are numerical requirements that government agencies need to achieve on an ongoing basis. The key performance indicator of the PSB is usually based on the number of PWUD arrested and referred to compulsory treatment centres. This policy penalizes police officers who refer PWUD to CDDT instead of arresting them, resulting in a fundamentally tense relationship between police and PWUD. To achieve their targets, police officers make arrests in the vicinity of, or right outside of, MMT clinics. As one PWUD informant disclosed:

Sometimes police go to the clinic to catch people for a urine test. It is scary. I would not go to drink methadone during that time. I have to go back to heroin under that situation. There is no other solution.

As a result of the tension and suspicion between police and PWUD, many PWUD respondents expressed negative attitudes towards police, using phrases such as “distrust,” “scary” and “as terrifying as a mouse confronting a cat” to describe their relationship with the police. A PWUD informant explained that the police “see us as social trash. We are labelled, and we are bad people forever, without dignity, not even treated as human beings.”

At a much more fundamental level, it was clear that the performance targets of the police were all grounded in punitive indicators, with no reference to any health-enabling indicators. This was in direct contrast to evaluation measures for the CDC, which were essentially based on health outcomes. Fears of and negative attitudes towards police by PWUD influenced treatment outcomes: 11 of the 16 clients interviewed expressed that they were unwilling to participate in CDDT because they did not want to go to police stations for urine tests (a positive result can lead to arrest): “I don't want to see any of them [police].” This unwillingness to participate in CDDT not only affects the health outcomes of the PWUD but also the performance targets of the CDC, which include adherence to MMT, as well as HIV prevention. One CDC official described the situation as follows:

We have our own performance indicators to achieve. We need to keep certain numbers of PWUD in the MMT clinic; administer HIV, hepatitis C and tuberculosis tests among them; and keep the HIV-positive rate as low as possible. Police arrests in the clinic make people afraid to come. Once they drop out of MMT, it is easy for them to get infected with HIV, as they have to use heroin. We in the CDC have to take responsibility because HIV prevalence is our performance indicator. This is pretty unfair.

In theory and based on existing policy, these agencies are meant to work cooperatively but in practice, the National Narcotics Control Commission (NNCC) is largely absent and PSBs are primarily responsible for implementing the drug control laws at local levels. This results in the dominance of the PSB in determining the fate of arrested PWUD. The performance evaluations of the PSB and the CDC being opposed undermines the goal of community-based treatment.

### Preliminary evidence for successful aspects of collaboration: take-home MMT

The Ping An Centre No. 1 selected and proposed MMT as the starting point to initiate institutional cooperation between CDC and PSB for two reasons. According to MMT participants, take-home methadone is one of their primary needs. Methadone is currently under strict management and clients are not allowed to take it outside of the clinic. However, a Ping An Centre No. 1 staff member indicated the following problem with daily clinic visits:

Daily visits prevent clients from living normal and productive lives, because they have to be late for their job or leave earlier. This may also disclose their drug use history to their employers and result in [PWUD] losing their jobs. This can even cause relapse to heroin.

Ping An Centre No. 1 petitioned permission from the Yunnan Institute for Drug Abuse and negotiated with the CDC and PSB to launch a take-home MMT programme. Although the PSB was concerned about the supply of methadone in the black market, drug safety was the primary concern for the CDC and officials worried especially about methadone being reached by children. To address this concern, Ping An Centre No. 1 clinic staff designed a pin-locked take-home box and MMT participants receive passwords through daily text messages. This design was the product of two months of ongoing discussion among all stakeholders about the equipment, the concerns, the risks, the needs of PWUD and consensus that even black market methadone can help to reduce heroin injection and contribute to HIV prevention. The boxes also have the option for GPS tracking, which provides reassurance to the police that the methadone boxes will stay within the homes of PWUD. A Ping An Centre No. 1 staff member noted initial positive cooperation outcomes from the take-home MMT programme:

Though very initial, MMT is already a platform for cooperation [between the CDC and PSB]. It is a state-sponsored programme and a crucial part of the national movement of HIV/AIDS prevention. Therefore, [it is] easier to start from here [MMT] to draw everyone into the conversation [of collaboration on CDDT].

At the time of writing, 72 PWUD were using this take-home methadone service. They were taking two to six days’ dosage of methadone at home, depending on their adherence records and ongoing urine test results. Preliminary results indicated that this service resulted in higher adherence rates and lower relapse rates, which enhanced the police's confidence in non-compulsory approaches to deal with PWUD [23]. Both Ping An Centre No. 1 staff and local police now agree that take-home methadone approach relieves the burden on police officers. A Ping An Centre No. 1 staff member shared the following: “Now, the police even take the initiative to lower the threshold for clients to be eligible to take methadone home.” A police officer commented that this change in threshold was required “to meet their needs, [which] also makes our work easier.” For their part, PWUD participants are motivated to adhere to in-clinic MMT to qualify for the take-home methadone service. A full analysis of the take-home service is currently underway.

## Discussion

Through semi-structured interviews of the PSB, CDC and Ping An Centre No. 1 staff and clients, we identified a need for general collaboration and shared goals with regard to the establishment, organization and execution of CDDT. Two related barriers to community-based treatment success emerged from the interview data: divergence in attitudes towards drug use, harm reduction and PWUD between local police and health officials (CDC officials and Ping An Centre No. 1 staff) and conflicting performance targets of the police and the CDC. Both of these barriers undermine the shared goal of treatment. There is a need to promote effective collaboration that serves the needs and treatment of PWUD; we identified the take-home MMT service (a product of collaboration among all stakeholders) as a promising model for future collaborations among governmental agencies at Ping An Centre No. 1 and other CDDT programmes in China.

These themes are common to other studies of the barriers to implementing community-based treatment in China that cite the lack of the necessary community infrastructure and the inadequacy of multi-agency cooperation. Harm reduction programming has long been confronted by opposing philosophical ideals and responses: one based on abstinence from and authoritarian criminalization of drug use, and the other based on empowering approaches that enable PWUD to take ownership of their health and eventually overcome drug dependence [9,12]. Yet, in China, the movement towards the latter is additionally hindered by a fragmented bureaucratic apparatus [24], which limits practical cooperation. It is also hampered by competing agendas and asymmetrical power dynamics of the different state agencies whose mandates intersect with drug use and PWUD, as our findings suggest.

As noted here and elsewhere [13], the local police maintain punitive approaches, which are ineffective and laden with abuses of the rights of PWUD [7]. Yet evidence suggests that a voluntary, community-based treatment approach is increasingly preferable, as it enhances people-centred care [25–27]. Annual targets related to crime and drug use are one of the most important indicators adopted by the higher-level government to evaluate the performance of local police officers. Failing to meet the annual target requirements could severely affect the salary and political career of both PSB and CDC staff. Competition for the same “targets” (PWUD) not only raises conflict between state agencies, but also prevents PWUD from seeking and adhering to treatment, which may not only affect their drug dependence, but can also negatively affect health and HIV-related outcomes.

As a result, a fundamental shift of the performance indicators in the police force is required. These indicators need to focus on the contribution of the PSB towards voluntary community-based treatment. Ideally, the PSB and CDC could agree to refer people arrested for drug use to community-based treatment centres. In this way, referral to treatment and MMT adherence could act as performance indicators for both the police and CDC, further strengthening collaboration and shared goals and potentially reducing criminal behaviour in China [28].

However, this approach would require a lengthy approval process before local police officers can start practising it. In 2014 and 2015, initial sensitization workshops on harm reduction were made available to top officials of the PSB and CDC, in combination with site visits to Ping An Centre No. 1 to facilitate observation of the take-home methadone programme and interaction with PWUD. This step achieved some convergence towards the shared goal of harm reduction, and representatives from both parties agreed that any PWUD who had completed compulsory treatment could be referred to Ping An Centre No. 1. Both the PSB and CDC officials saw ways in which they could work together to control drug use locally. The PSB agreed to make harm reduction training a standard practice for all the district's police stations. These workshops on harm reduction are designed to facilitate a shift from punitive practices to a community-based approach that is evidence-based, humanistic and less restrictive, which has been demonstrated to be successful in other countries [27,29].

We argue that instead of perfecting the details of articles in the law, as some studies suggest [30,31], a more cost-efficient way to practise CDDT in China is to first illuminate workable models in particular local settings, which would provide both evidence and impetus to amend the law, and to indicate the resources required. Hence it would be valuable to adopt a bottom-up approach to identifying and piloting solutions that are specific in local situations. These solutions ought to be developed by local agencies, but still operate under the overall goals of harm reduction nationally [4,32] and the general guidelines of the inter-agency collaboration. To illustrate this point, we note that although the 2008 drug control law recommended non-compulsory measures, it vaguely placed responsibility for providing community-based treatment on the powerless and resource-deficient sub-district administrative agencies. These agencies have not yet implemented community-based treatment, thereby perpetuating the default detention approaches. The collaborative development of the take-home MMT programme described in this article demonstrates that different governmental agencies can successfully and collaboratively serve PWUD in spite of power imbalances that exist among PWUD, local police, CDC officials and treatment centre staff. This is a workable model demonstrating collaboration with a common harm reduction goal, with an emphasis on improving the relationship between police and PWUD, that can potentially be scaled up based on evidence of its effectiveness.

Given the importance of drug use in driving the HIV epidemic in China [33], collaboration and the shift in policing indicators and approaches becomes even more important considering the policy goals of halting China's HIV epidemic among PWUD [4,32]. MMT is an effective entry point for increasing early testing of HIV and linking to antiretroviral therapy [34,35], both of which are poorly available to PWUD in China and globally [11]. However, given our findings showing that PWUD avoid accessing MMT for fear of arrests, the potential for pairing HIV-related services with MMT is unrealized. Other studies have demonstrated the negative impact of potential detention on healthcare-seeking behaviours among PWUD [36].

Finally, community-based treatment facilities need to both medically serve PWUD and simultaneously act as a mediator between PWUD and government agencies, especially the police. Trust from PWUD is the most valuable social capital CDDT programmes can achieve. To reach a consensus on supporting a community-based treatment model while accounting for the needs of all parties, persistent negotiations on both the individual level and on the institutional level need to be carried out. The former happens through interpersonal interaction among local leaders (both PSB and CDC) and PWUD, mainly through informal meetings individually, and occasional formal meetings when necessary. The latter includes holding multi-agency meetings, providing training to police and arranging study visits for top officials. To promote these goals, the Ping An Centre No. 1 arranged study visits to Seattle, WA, USA (for both PSB and CDC top officials) and India (for CDC officials) to experience people-centred and voluntary harm reduction approaches. These visits also provided senior officials with opportunities to witness the result of cooperation among multiple stakeholders and to investigate the possibility of non-compulsory measures in the Chinese context. Commenting on this experience, a PSB participant noted, “it is surprising that police and PWUD can become friends in Seattle. It [a community-based, non-compulsory treatment model] is worth trying.”

### Limitations

Before firm conclusions can be made from this paper, we highlight that limitations related to participant sampling limits the extent to which our claims can be generalized [21]. Nevertheless, our intention was to provide the perspectives of a few participants in a specific context through qualitative interviews [37], focussing on representativeness rather than ability to generalize [21,38]. Additional data from the take-home methadone centre are currently being analyzed and will provide a more in-depth analysis of the broad outcomes of such an approach for collaboration across different stakeholders in harm reduction.

## Conclusions

The early success of the take-home MMT programme demonstrates that effective collaboration is possible among all stakeholders concerned with drug use, HIV and harm reduction in this setting. This is consistent with the United Nations Office of Drug Control, which specifies that treatment needs to “respond to the needs and resources of communities” and to “mobilise all available resources in the community to meet their clients’ needs” and to “work with law enforcers” [8]. In the Ping An Centre No. 1 project, clients’ primary need of taking methadone home has been addressed, and initial cooperation between health department and law enforcement has been achieved to provide harm reduction services for PWUD. However, we identified persistent challenges associated with differences in attitudes towards PWUD held by police and health officials. These differences are compounded by the conflicting performance targets of the PSB and CDC. Our findings suggest a great need for cooperation among all stakeholders. It is especially important to provide police with workable models that could help develop political support for the community-based treatment model. Considering that PWUD remain one of the highest-risk populations for HIV infection in China and current practices of compulsory detainment of PWUD are not effective, it is crucial to address the tensions and conflicts among the stakeholders for effective implementation of CDDT.
